# The metabolic score for insulin resistance index is associated with myocardial ischemia, with non-alcoholic fatty liver disease serving as an important bridge

**DOI:** 10.3389/fendo.2025.1592463

**Published:** 2025-06-06

**Authors:** Yu Tian, Fan Sun, Wenji Yu, Feifei Zhang, Jianfeng Wang, Xiaoliang Shao, Bao Liu, Xiaoyu Yang, Yongjun Chen, Sijin Li, Yuetao Wang

**Affiliations:** ^1^ Department of Nuclear Medicine, The Third Affiliated Hospital of Soochow University, Changzhou, Jiangsu, China; ^2^ Institute of Clinical Translation of Nuclear Medicine and Molecular Imaging, Soochow University, Changzhou, Jiangsu, China; ^3^ Department of Cardiology, The Third Affiliated Hospital of Soochow University, Changzhou, Jiangsu, China; ^4^ Department of Nuclear Medicine, First Hospital of Shanxi Medical University, Taiyuan, Shanxi, China

**Keywords:** metabolic score for insulin resistance (METS-IR), non-alcoholic fatty liver disease (NAFLD), myocardial ischemia, mediating effect, myocardial perfusion imaging (MPI)

## Abstract

**Background:**

This study aimed to evaluate the correlation between the metabolic score for insulin resistance (METS-IR) and myocardial ischemia based on myocardial perfusion imaging (MPI) and further examine whether non-alcoholic fatty liver disease (NAFLD) has a potential role in mediating these associations.

**Methods:**

This retrospective study enrolled 1,242 patients with suspected coronary artery disease (CAD) who underwent single-photon emission computed tomography myocardial perfusion imaging (SPECT-MPI) at the Third Affiliated Hospital of Soochow University from 1 January 2022 to 31 December 2024. The association between METS-IR and myocardial ischemia was analyzed using the logistic regression model. The mediating effect of NAFLD was evaluated through mediation analysis to explore the potential mechanism underlying the association between METS-IR and myocardial ischemia.

**Results:**

The final group of participants included 335 patients; 179 (53.4%) patients had myocardial ischemia. Overall, the mean age was 61.45 ± 10.20 years, 188 (56.1%) were men, and the mean body mass index was 24.69 ± 3.21 kg/m^2^. Mean METS-IR was 2.40 ± 0.22 (range, 1.79–3.80). The results of the single-factor analysis showed that a per-SD increase in METS-IR was independently associated with myocardial ischemia (OR, 3.92; 95% CI: 1.38-11.08; *P* =0.010). After adjusting for all interfering factors, METS-IR had no associations with myocardial ischemia. The results of the mediation analysis show that NAFLD is a complete mediator variable between MERSIR and myocardial ischemia.

**Conclusion:**

This study provides evidence for the relationship between METS-IR and myocardial ischemia and highlights the important mediating role of NAFLD in this relationship.

## Introduction

Cardiometabolic diseases (CMDs) include ischaemic heart disease (IHD), type 2 diabetes (T2D), and stroke (1). Globally, CMDs continue to account for the highest number of deaths and hospitalizations, especially, IHD is the main cause of morbidity and mortality (2). Myocardial ischemia serves as a robust indicator of IHD and is strongly associated with an elevated risk of cardiovascular mortality. Single-photon emission computed tomography myocardial perfusion imaging (SPECT-MPI) is a highly accurate, evidence-based non-invasive imaging method for diagnosing myocardial ischemia (3). Large cohort studies have demonstrated that the presence of myocardial ischemia is independently associated with an increased incidence of major adverse cardiovascular events (MACEs) (4, 5). Therefore, identifying modifiable risk factors within the population, particularly those related to cardiometabolic diseases, is crucial for the precise prevention and treatment of cardiometabolic disease and the reduction of cardiovascular mortality.

Insulin resistance (IR) is known to exist in the early stages of various chronic conditions, including hypertension, metabolic syndrome (MS), T2D, and chronic kidney disease (CKD) (6). Non-alcoholic fatty liver disease (NAFLD), a manifestation of MS, frequently co-occurs alongside prevalent metabolic disorders including obesity, hypertension, dyslipidemia, and diabetes mellitus. NAFLD shares common pathophysiological mechanisms with cardiometabolic diseases, such as IR, endothelial dysfunction, oxidative stress (OS), and systemic inflammation (7). A large-scale study showed that major alterations in human metabolism may begin long before the clinical onset of IHD (8). IR is an important factor in the development of ischemic heart disease and predisposes individuals to microvascular and macrovascular complications associated with diabetes and metabolic syndrome (9). IR is characterized by a diminished responsiveness of target tissues to the normal circulating levels of insulin. This results in ineffective glucose transport into target cells, which in turn leads to the development of metabolic abnormalities such as hyperglycemia (10). IR plays a significant role in the development and prognosis of various cardiovascular diseases (CVDs). A study has shown that fasting insulin levels are positively correlated with adverse echocardiographic features, and they increase the risk of patients developing heart failure (HF) (11).

In recent years, the metabolic score for insulin resistance (METS-IR), a novel non-insulin-based index of fasting IR (12), has garnered increasing attention. Its popularity has grown due to its non-invasive nature and the simplicity of its calculation. Some researchers have demonstrated that certain non-insulin IR indicators, such as the triglyceride-to-high-density lipoprotein cholesterol ratio (TG/HDL-C) and the triglyceride-glucose (TyG) index, are associated with IHD (13, 14). A study indicated that an elevated METS-IR score predicted future IHD in non-diabetic community-dwelling Koreans more effectively than metabolic syndrome did, making it a valuable predictive indicator (15). However, few studies have explored the relationship between the METS-IR and myocardial ischemia.

Therefore, the primary objective of this study was to evaluate the relationship between the METS-IR and myocardial ischemia based on SPECT-MPI. Additionally, the study aimed to investigate whether NAFLD plays a potential mediating role in this association.

## Methods

### Study design and population

**Figure 1:**
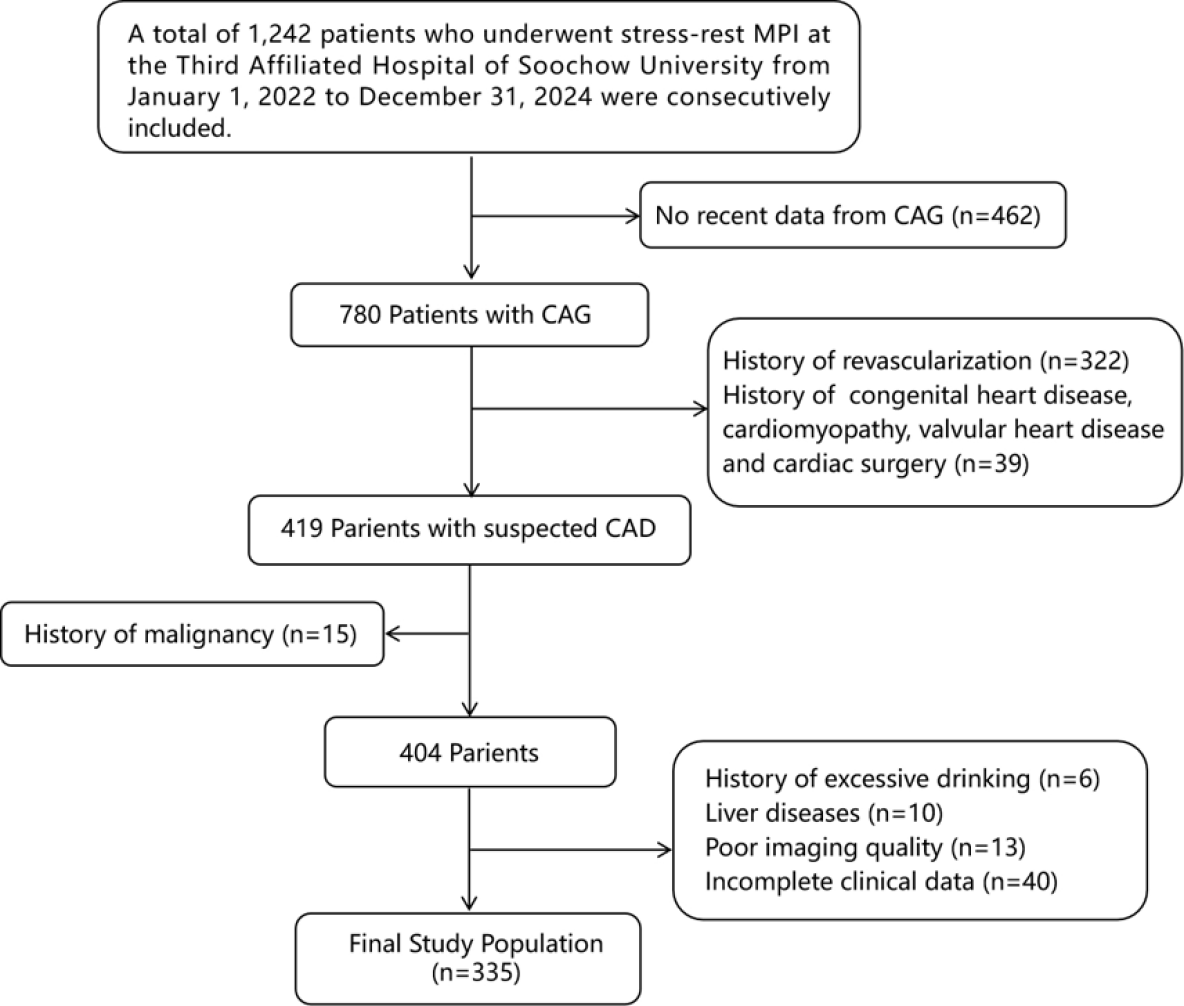
Flowchart of participants inclusion. MPI, myocardial perfusion imaging; CAD, coronary artery disease; CAG, coronary angiography.

In this retrospective cohort study, 1,242 suspected coronary artery disease (CAD) patients who underwent stress-rest myocardial perfusion imaging (MPI) at the Third Affiliated Hospital of Soochow University from 1 January 2022 to 31 December 2024 were consecutively included. To eliminate the influence of obstructive CAD on myocardial ischemia, all the patients needed to have coronary angiography (CAG) results from within 3 months and have undergone a blood test within 1 week. The exclusion criteria were as follows (1): history of revascularization (2); patients with acute coronary syndrome (ACS) or prior myocardial infarction (3); history of congenital heart disease (4); history of cardiomyopathy (5); severe valvular heart disease or post-valve replacement surgery (6); history of malignancy or history of radiotherapy/chemotherapy (7); long-term heavy alcohol consumption or history of excessive drinking (≥40g/day for men, ≥20g/day for women) (8); patients with previous viral hepatitis, abnormal liver function, or cirrhosis (9); poor imaging quality (10); missing lab values or METS-IR components. **Figure 1** shows the flowchart of population selection.

Traditional cardiovascular disease risk factors were collected by comprehensively querying the hospital’s medical records system. These included body mass index (BMI), hypertension, hyperlipidemia, diabetes mellitus (DM), and smoking history. Height and weight were measured to calculate BMI. Abdominal obesity was assessed using waist circumference (≥90 cm for men, ≥85 cm for women) as the standard (16). Hypertension was defined as a systolic blood pressure ≥140 mmHg, a diastolic blood pressure ≥90 mmHg, or the use of antihypertensive medication. Hyperlipidemia was defined as a history of hyperlipidemia, total cholesterol >5.2 mmol/L, low-density lipoprotein cholesterol >3.36 mmol/L, or the use of lipid-lowering medications. DM was defined as self-reported DM, antidiabetic medication use, or fasting glucose ≥7.0 mmol/L (17). Current smoking was defined as smoking within the previous 6 months. Blood test indicators collected within 1 week of examination included fasting blood glucose (FBG), total cholesterol (TC), triglycerides (TG), high-density lipoprotein cholesterol (HDL-C), low-density lipoprotein cholesterol (LDL-C), creatinine, and uric acid (UA). At the same time, clinical medication data were collected, including statins, SGLT2 inhibitors, metformin, and other drugs that affect insulin metabolism. The research protocol was approved by the institutional review board or ethics committee.

### Assessment of METS-IR

The METS-IR was calculated using the following formula: METS-IR = {Ln [(2 × fasting glucose (mg/dL)) +fasting TG (mg/dL)] × BMI (kg/m2)}/{Ln [HDL-C (mg/dL)]} (12).

METS-IR was treated as a continuous exposure variable in our study and all enrolled participants were stratified into tertiles based on METS-IR values for subsequent analyses.

### SPECT/CT image acquisition

Before the examination, the subjects were instructed to discontinue medications that affect heart rate or vasodilation, such as β-blockers, nitrates, and calcium channel blockers for 24–48 hours, and to avoid caffeine-containing foods or drinks for at least 24 hours. All the subjects underwent a 2-day gated stress (including 99% exercise stress and 1% adenosine drug stress) and rest myocardial perfusion imaging. Images were acquired using a dual-probe SPECT/CT system (T16 model, Siemens, Germany) 60 to 90 minutes after intravenous injection of ^99m^Tc-MIBI (radiochemical purity > 95%, injected dose of 740–925 MBq). The specific parameters were as follows: magnification factor 1.45, matrix size 128×128, energy peak 140 keV, and a 20% window width. Using ECG gating technology, 8 frames were acquired for each R-R interval. The projection data were filtered using the 3D Flash iterative method (with 16 iterations and 2 subsets), resulting in the three-dimensional axial images of the left ventricle (short axis, horizontal long axis, and vertical long axis). The image acquisition programs all followed the suggestions of the relevant guidelines (18, 19).

After completing the MPI acquisition, low-dose CT was performed to obtain data for coronary artery calcium score (CACS), epicardial fat volume (EFV), and NAFLD. The parameters were as follows: tube voltage of 120 KV, tube current of 100 mA, and thickness of 3 mm. The scanning range was from the plane below the tracheal carina to 1–2 cm below the diaphragm surface of the heart. The scan was completed with one breath-hold after inhalation, and the scanning time was 8–13 seconds. Data were collected between 60% and 80% of the R-R interval.

### Imaging data processing and analysis

#### Definition of myocardial ischemia

SPECT-MPI images were reconstructed and analyzed using dedicated software, and the reconstructed stress-rest myocardial perfusion images were independently analyzed by two experienced nuclear medicine physicians. In the case of differing opinions, a third senior physician participated in the review, and a consensus diagnosis was made after discussion. Attenuation correction was applied to the patients exhibiting artifacts in the inferior and anterior walls. Perfusion images were analyzed using a 17-segment model with a five-point continuous scoring system (0, normal; 1, mildly abnormal; 2, moderately abnormal; 3, severely abnormal; 4, absence of segmental uptake). The summed stress score (SSS) and summed rest score (SRS) were calculated from all the abnormal segments in the stress and rest data. The summed difference score (SDS) was calculated by subtracting the SRS from the SSS. Myocardial ischemia was defined as a reversible perfusion defect with a SDS ≥ 2 (20, 21).

#### Definition of NAFLD

In the right lobe of the liver, two regions of interest (ROIs) were selected, and one ROI was selected in the spleen at the same level. The area of the ROI was greater than 100 cubic millimeters (mm³). The mean values were then calculated to determine the liver/spleen CT density [Hounsfield units (HU)] ratio. NAFLD was defined as having a mean liver/spleen CT density ratio <1 or a mean liver CT value ≤40 HU with no personal history of alcohol use/abuse and no history of liver disease (22).

### Coronary artery calcium score

CAC was identified as a dense area in the coronary artery that surpassed the threshold of 130 HU. Using the Agatston automatic analysis software for measurement, the sum of the calcium scores of the left main coronary artery (LM), left anterior descending coronary artery (LAD), left circumflex coronary artery (LCX), and right coronary artery (RCA) constitutes the CACS (23). The CACS was then categorized into two groups: no calcification (score = 0) and presence of calcification (score > 0). Calcification in the diagonal branches was included in the LAD score, while calcification in the obtuse marginal branches was included in the LCX score for calculation purposes.

### Epicardial fat volume

On consecutive axial images, the adipose tissue area between the myocardium and the pericardium was manually delineated (with a predefined threshold of -190 to -30 HU). The ROI extended from the upper boundary at the pulmonary artery bifurcation to the lower boundary at the base of the heart. The EFV (cm³) and fat attenuation value (FAV) were calculated using semi-automatic measurement software, Syngo Volume (24).

### Evaluation of obstructive CAD

CAG was conducted via radial or femoral artery puncture using the Seldinger technique and the Judkins method. The luminal diameter was independently assessed by two experienced cardiologists. In cases of disagreement, a third cardiologist was involved to reach a consensus. The primary focus was evaluating the degree of stenosis in the LM, LAD, LCX, and RCA. Obstructive CAD was defined as ≥50% stenosis in at least one coronary artery and without obstructive CAD was defined as coronary artery stenosis of less than 50% (including 0% stenosis) (25)

### Statistical analysis

All statistical analyses were conducted using R software (version 4.2.2), EmpowerStats (version 2.0), and the rms package. The normality of continuous variables was assessed via the Kolmogorov–Smirnov test. Continuous variables were reported as mean ± standard deviation (SD) if normally distributed, or as median with interquartile range (IQR; 25th to 75th percentile) for non-normal distributions. Baseline characteristics were compared between patients with and without myocardial ischemia. For normally distributed data, independent t-tests were employed, whereas the Mann–Whitney U test was used for non-normally distributed data to evaluate the differences in the quantitative variables between two groups. Categorical variables were presented as counts (%) and analyzed using chi-square tests or Fisher’s exact tests, as appropriate. In the univariate analyses, Pearson correlation coefficients were calculated for continuous variables, while Spearman correlation coefficients were used for categorical variables. The impact of factors associated with tertiles of METS-IR was evaluated using trend tests.

To evaluate the association between METS-IR and myocardial ischemia, we employed multivariable logistic regression models. Three distinct models were developed (1): model 1, an univariable model with METS-IR as a predictor (2); model 2, adjusted for age and gender (3); model 3, we assessed the confounding effect of covariates by examining the association of METS-IR with myocardial ischemia before and after adding the covariate. If the scale of the β coefficient changed by >10%, we concluded that the covariate was a confounder. Furthermore, any variable that was significantly different between the myocardial ischemia and non-ischemia groups (*P*<0.10) was also retained as confounder. Based on a systematic review of the existing literature, this study included age, gender, hypertension, diabetes, hyperlipidemia, current smoking, EFV, CACS, and obstructive CAD in the adjustment model for correction. Considering the previous literature reporting a possible association between IR and NAFLD, fatty liver was not included as a variable for model adjustment. The results from these models are presented as odds ratios (ORs) with corresponding 95% confidence intervals (CIs).

Moreover, a mediation analysis was conducted to examine the link between NAFLD, METS-IR, and myocardial ischemia. This analysis was adjusted for various factors, including gender, age, smoking status, history of hypertension, DM, hyperlipidemia, EFV, CACS, and obstructive CAD. The presence of a mediating effect was defined as satisfying all the following conditions: having a significant indirect effect, a significant total effect, and a positive proportion of the mediator effect. 

## Results

### Patient characteristics

**Figure 2:**
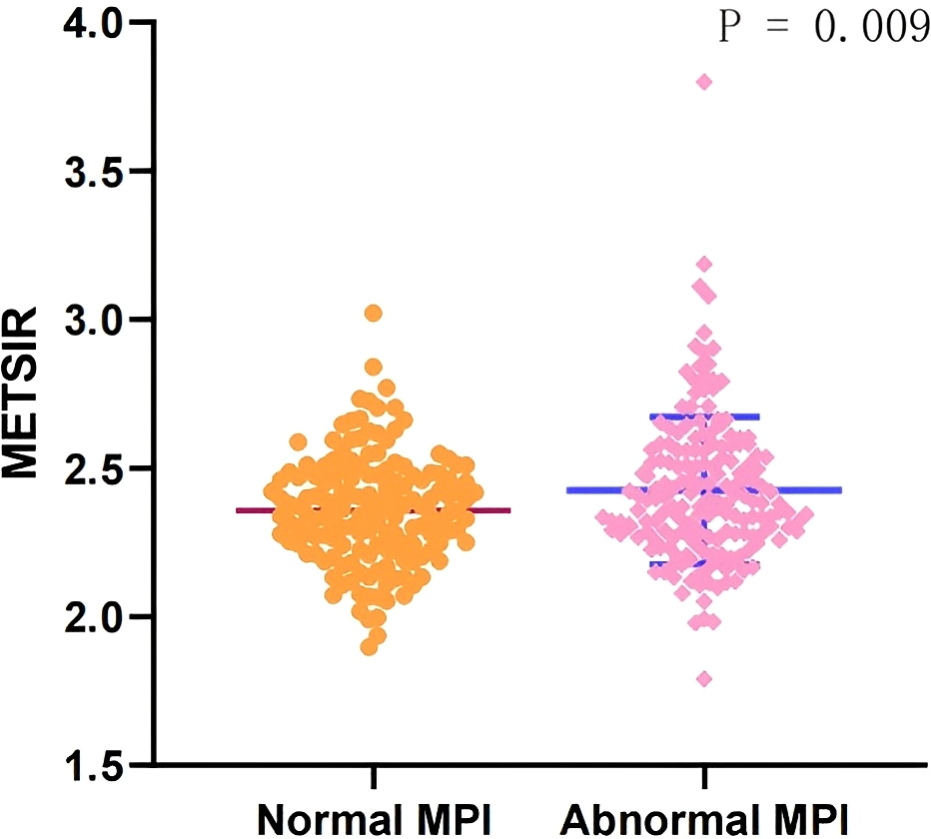
Comparison of the METS-IR index in patients with normal MPI and abnormal MPI. METS-IR, Metabolic Score for Insulin Resistance; MPI, myocardial perfusion imaging.

The baseline characteristics of all the patients are shown in **Table 1**, and 179 (53.4%) patients had myocardial ischemia. Overall, the mean age was 61.45 ± 10.20 years, 188 (56.1%) were men, and the mean BMI was 24.69 ± 3.21 kg/m^2^. The mean METS-IR was 2.40 ± 0.22 (range, 1.79–3.80). Compared with the patients without myocardial ischemia, the patients with myocardial ischemia tended to have higher BMI (25.13 ± 3.29 kg/m^2^ vs. 24.19 ± 3.04 kg/m^2^) and METS-IR (2.42 ± 0.25 vs. 2.36 ± 0.19, **Figure 2**), and a higher proportion of NAFLD and a lower age (all *P <*0.05).

**Table 1:** Baseline patient characteristics.

Variable	Total	Myocardial ischemia	Non-ischemia	*P*-value
	(n=335)	(n=179)	(n=156)	
Age, years	61.45 ± 10.20	60.25 ± 10.75	62.83 ± 9.37	0.021*
Male, n (%)	188 (56.1%)	103 (57.5%)	85 (54.5%)	0.574
Abdominal obesity, n (%)	217 (64.8%)	118 (65.9%)	99 (63.5%)	0.638
Current smoking, n (%)	117 (34.9%)	72 (40.2%)	45 (28.9%)	0.067
Hypertension, n (%)	219 (65.4%)	125 (69.8%)	94 (60.3%)	0.066
Hyperlipidemias, n (%)	138 (41.2%)	75 (41.9%)	63 (40.4%)	0.779
DM, n (%)	88 (26.3)	54 (30.2%)	34 (21.8%)	0.082
BMI, kg/m²	24.69 ± 3.21	25.13 ± 3.29	24.19 ± 3.04	0.007*
Imaging				
NAFLD, n (%)	124 (37%)	93 (52.0%)	31 (19.9%)	<0.001*
EFV, cm3	129.91 ± 47.32	134.36 ± 50.56	124.79 ± 42.90	0.072
FAV, HU	-76.67 ± 3.81	-76.53 ± 3.82	-76.81 ± 3.81	0.397
CACS, n (%)				0.434
0	147 (43.9%)	75 (41.9%)	72 (46.2%)	
>0	188 (56.1%)	104 (58.1%)	84 (53.9%)	
Obstructive CAD, n (%)				0.296
No	153 (45.7%)	77 (43.0%)	76 (48.7%)	
Yes	182 (54.3%)	102 (57.0%)	80 (51.3%)	
Blood tests				
FBG, mg/dL	99.47 (90.10,122.63)	101.27 (90.10,122.63)	97.85 (88.21,113.35)	0.060
TC, mg/dL	154.81± 41.49	156.00 ± 41.35	153.44 ± 41.74	0.574
TG, mg/dL	123.08 (88.55,170.45)	122.19 (89.43,178.42)	123.08 (86.55,166.46)	0.050
HDL-C, mg/dL	46.14 ± 11.28	45.51 ± 11.79	46.87 ± 10.65	0.466
LDL-C, mg/dL	87.11 ± 32.40	87.96 ± 32.19	86.13 ± 32.71	0.566
Creatinine, µmol/L	67.97 ± 17.79	68.32 ± 20.41	67.56 ± 14.22	0.571
UA, µmol/L	331.75 ± 87.19	336.10 ± 88.58	326.73 ± 85.56	0.328
METS-IR	2.40 ± 0.22	2.42 ± 0.25	2.36 ± 0.19	0.009*
Lipid-lowering drugs				0.759
Statins (%)	266 (79.4%)	141 (78.8%)	125 (80.1%)	
Fibrates (%)	25 (7.0%)	14 (7.8%)	11 (7.1%)	
Hypoglycemic agents (%)	130 (38.8)	68 (38.0%)	62 (39.7%)	0.742
SGLT-2 (%)	16 (4.8%)	4 (2.2%)	12 (7.7%)	
Metformin (%)	28 (8.4%)	12 (6.7%)	16 (10.3%)	

DM, diabetes mellitus; BMI, body mass index; NAFLD, non-alcoholic fatty liver disease; EFV, epicardial fat volume; FAV, fat attenuation value; CACS, coronary artery calcification score; CAD, coronary artery disease; FBG, fasting blood glucose; TC, total cholesterol; TG, triglyceride; HDL-C, high‐density lipoprotein cholesterol; LDL-C, low‐density lipoprotein cholesterol; UA, uric acid; METS-IR, Metabolic Score for Insulin Resistance; SGLT-2, sodium/glucose cotransporter 2. *P<0.05

**Table 2** presents the characteristics of the participants with different METS-IR index tertiles. Significant statistical differences were detected in age, EFV, FAV, TC, creatinine, UA, and smoking, and for the incidence of abdominal obesity, hypertension, hyperlipidemia, DM, and NAFLD among the subgroups for the tertiles of the METS-IR index (*P* for trend <0.05). Although the ischemia rate increased across METS-IR tertiles, the trend was not statistically significant (P = 0.251).

**Table 2:** Baseline characteristics stratified by tertiles of METS-IR.

Characteristic	Tertile 1	Tertile 2	Tertile 3	*P*-value
	(n=112)	(n=111)	(n=112)	
Age, years	62.00 ± 10.77	62.90 ± 9.52	59.46 ± 10.04	0.033*
Male, n (%)	60 (53.6%)	61 (55.0%)	67 (59.8%)	0.613
Abdominal obesity, n (%)	57 (50.9%)	72 (64.9%)	88 (78.6%)	<0.001*
Current smoking, n (%)	29 (25.9%)	36 (32.4%)	52 (46.4%)	0.023*
Hypertension, n (%)	60 (53.6%)	71 (64.0%)	88 (78.6%)	<0.001*
Hyperlipidemias, n (%)	39 (34.8%)	39 (35.1%)	60 (53.6%)	0.005*
DM, n (%)	16 (14.3%)	29 (26.1%)	43 (38.4%)	<0.001*
Myocardial ischemia, n (%)	56 (50.0%)	56 (50.5%)	67 (59.8%)	0.251
NAFLD, n (%)	21 (18.8%)	39 (35.1%)	64 (57.1%)	<0.001*
EFV, cm3	110.72 ± 40.86	133.51 ± 46.02	145.91 ± 48.28	<0.001*
FAV, HU	-75.57 ± 4.11	-77.74 ± 3.55	-76.72 ± 3.45	<0.001*
CACS, n (%)				0.594
0	53 (47.3%)	45 (40.5%)	49 (43.8%)	
>0	59 (52.7%)	66 (59.5%)	63 (56.2%)	
Obstructive CAD, n (%)				0.1
No	59 (52.7%)	51 (45.9%)	43 (38.4%)	
Yes	53 (47.3%)	60 (54.1%)	69 (61.6%)	
TC, mg/dL	162.92 ± 42.29	148.27 ± 35.21	153.18 ± 45.28	0.027*
LDL-C, mg/dL	89.76 ± 35.53	83.32 ± 27.12	88.22 ± 33.83	0.302
Creatinine, µmol/L	63.99 ± 14.08	66.92 ± 14.39	72.96 ± 22.49	<0.001*
UA, µmol/L	315.15 ± 82.63	320.84 ± 78.68	359.01 ± 93.60	<0.001*

DM, diabetes mellitus; NAFLD, non-alcoholic fatty liver disease; EFV, epicardial fat volume; FAV, fat attenuation value; CACS, coronary artery calcification score; CAD, coronary artery disease; FBG, fasting blood glucose; TC, total cholesterol; LDL-C, low‐density lipoprotein cholesterol; UA, uric acid; METS-IR, Metabolic Score for Insulin Resistance. *P<0.05.

### Association of METS-IR with myocardial ischemia

**Table 3** displays the association of METS-IR with myocardial ischemia. In model 1, a per-SD increase in METS-IR was independently associated with myocardial ischemia (OR, 3.92; 95% CI: 1.38-11.08; *P* =0.010). In model 2, after adjusting for age and gender, significant associations were sustained in the myocardial ischemia group (OR, 3.67; 95% CI: 1.27-10.61; *P* =0.016). After adjusting for all interfering factors in model 3, METS-IR had no association with myocardial ischemia. However, in the three models, the ORs with corresponding CIs did not show positive significant correlations among T1, T2, and T3.

**Table 3:** The association between METS-IR and myocardial ischemia.

Variable	Model 1	*P*-value	Model 2	*P*-value	Model 3	*P*-value
	OR (95% CI)		OR (95% CI)		OR (95% CI)	
METS-IR	3.92 (1.38, 11.08)	0.01*	3.67 (1.27, 10.61)	0.016*	1.61 (0.46, 5.607)	0.458
Tertile 1	1		1		1	
Tertile 2	1.02 (0.60, 1.72)	0.946	1.00 (0.59, 1.70)	0.992	0.75 (0.42, 1.34)	0.334
Tertile 3	1.49 (0.88, 2.53)	0.14	1.43 (0.84, 2.45)	0.189	1.01 (0.50, 1.79)	0.864
p for trend	0.13		0.177		0.938	

Model 1 was not adjusted.

Model 2 was adjusted for gender and age.

Model 3 was adjusted for gender, age, smoking, hypertension, diabetes mellitus, hyperlipidemia, epicardial fat volume, coronary artery calcification score, obstructive coronary artery disease, and abdominal obesity.

METS-IR, Metabolic Score for Insulin Resistance; OR, odds ratio; CI, confidence interval. *P<0.05.

### Associations of NAFLD with METS-IR and myocardial ischemia

As **Table 4** shows, the univariate analysis indicated that BMI, current smoking, and NAFLD were related to myocardial ischemia (all *P* < 0.05). In the multivariate logistic regression analysis adjusted for traditional risk factors for myocardial ischemia (such as age, gender, smoking, hypertension, hyperlipidemia, and DM), CACS, EFV, and obstructive CAD, BMI (OR, 1.08, 95% CI: 1.00-1.17, *P* =0.041) and NAFLD (OR, 4.97, 95% CI: 2.68-9.24, *P <*0.001) were independent risk factors for myocardial ischemia.

**Table 4:** Association between myocardial ischemia and clinical risk factors in the patients.

Variable	Univariate analysis	P-value	Multivariate analysis	P-value
	OR (95% CI)		OR (95% CI)	
Age ≥ 60, years	0.79 (0.51, 1.24)	0.308		
Male, n (%)	1.13 (0.73, 1.74)	0.574		
BMI, kg/m²	1.10 (1.03, 1.18)	0.008	1.08 (1.00, 1.17)	0.041*
Abdominal obesity, n (%)	1.11 (0.71, 1.75)	0.638		
Current smoking, n (%)	1.60 (1.00, 2.55)	0.048*	1.50 (0.91 ~ 2.48)	0.109
Hypertension, n (%)	1.53 (0.97, 2.40)	0.067		
Hyperlipidemias, n (%)	1.06 (0.69, 1.65)	0.778		
DM, n (%)	1.55 (0.94, 2.55)	0.083		
NAFLD, n (%)	4.36 (2.67, 7.12)	<0.001*	4.33 (2.49, 7.54)	<0.001*
EFV, cm3	1.00 (1.00, 1.01)	0.073		
FAV	1.02 (0.96, 1.08)	0.511		
CACS>0, n (%)	1.19 (0.77, 1.83)	0.433		
Obstructive CAD, n (%)	1.26 (0.82, 1.94)	0.296		
FBG, mg/dL	1.10 (0.99, 1.23)	0.065		
TC, mg/dL	1.06 (0.87, 1.30)	0.573		
TG, mg/dL	1.23 (0.99, 1.52)	0.059		
HDL-C, mg/dL	1.07 (0.83, 1.38)	0.605		
LDL-C, mg/dL	0.66 (0.31, 1.39)	0.273		
Creatinine, µmol/L	1.00 (0.99, 1.01)	0.697		
UA, µmol/L	1.00 (1.00, 1.00)	0.327		
METS-IR	3.92 (1.38, 11.08)	0.01*	1.96 (0.58, 6.63)	0.281

DM, diabetes mellitus; BMI, body mass, index; NAFLD, nonalcoholic fatty liver disease; EFV, epicardial fat volume; FAV, Fat Attenuation Value; CACS, coronary artery calcification score; CAD, coronary artery disease; FBG, fasting blood glucose; TC, total cholesterol; TG, triglyceride; HDL-C, high‐density lipoprotein cholesterol; LDL-C, low‐density lipoprotein cholesterol; UA, uric acid; METS-IR, metabolic score for insulin resistance. *P<0.05

**Table 5** displays the association between METS-IR and NAFLD after multivariate logistic regression. After adjusting for all interfering factors, METS-IR was positively and significantly associated with NAFLD (OR, 20.23, 95% CI: 4.32-94.97, *P <*0.001).

**Table 5:** The association between METS-IR and NAFLD.

Variable	Model 1		Model 2		Model 3	
	OR (95% CI)	*P*-value	OR (95% CI)	*P*-value	OR (95% CI)	*P*-value
METS-IR	74.13 (19.48, 282.03)	<0.001*	73.23 (18.93, 283.32)	0.016*	20.23 (4.32, 94.71)	<0.001*
Tertile 1	1		1		1	
Tertile 2	2.35 (1.27, 4.34)	0.006*	2.32 (1.25, 4.28)	0.007*	2.05 (1.00, 4.21)	0.049*
Tertile 3	5.78 (3.16, 10.57)	<0.001*	5.59 (3.04, 10.28)	<0.001*	3.30 (1.57, 6.93)	0.001*
p for trend	<0.001*		<0.001*		<0.001*	

Model 1 was not adjusted.

Model 2 was adjusted for gender and age.

Model 3 was adjusted for gender, age, smoking, hypertension, diabetes mellitus, hyperlipidemia, epicardial fat volume, uric acid, total cholesterol, and abdominal obesity.

METS-IR, Metabolic Score for Insulin Resistance; NAFLD, non-alcoholic fatty liver disease; OR, odds ratio; CI, confidence interval. *P<0.05

### Mediating role of NAFLD

**Figure 3:**
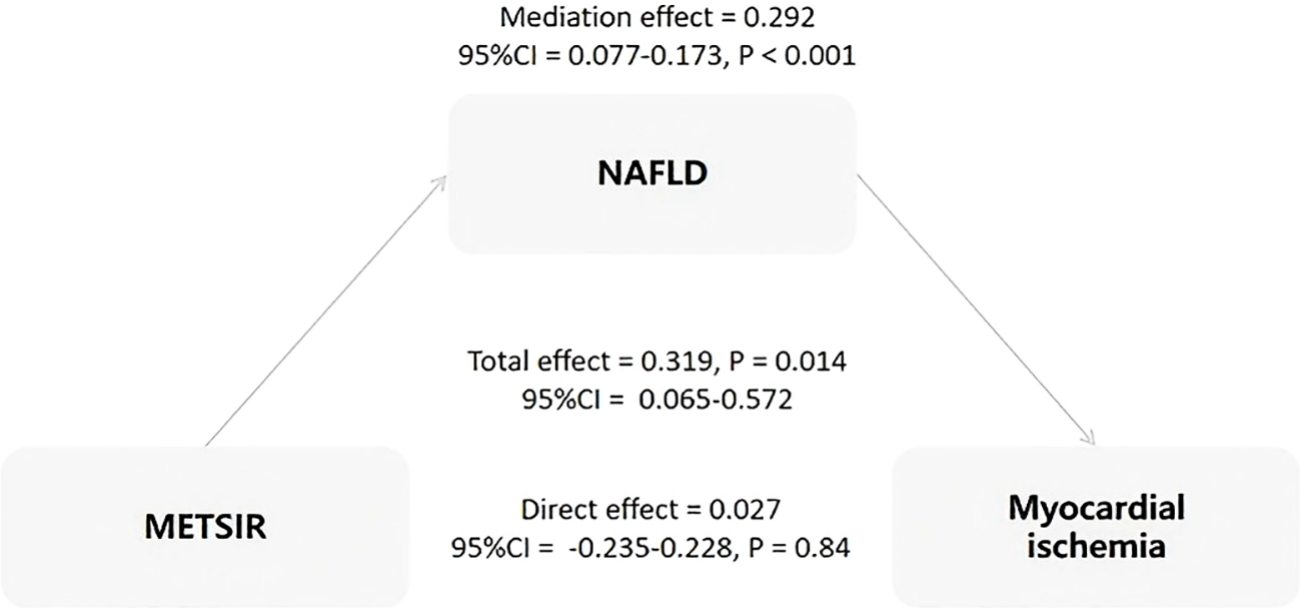
Analysis of the mediation effect of NAFLD on the association between METS-IR and myocardial ischemia. METS-IR, Metabolic Score for Insulin Resistance; NAFLD, non-alcoholic fatty liver disease; CI, confidence interval.

The results of the mediation analysis are shown in **Figure 3**. NAFLD was found to be a complete mediator variable between MERSIR and myocardial ischemia. Additionally, we assessed the mediating roles of other metabolism indicators, including DM and EFV, with no mediating effect found for any of them.

## Discussion

The principal findings of the present study were as follows: (I) Compared with non-myocardial ischemia patients, patients with myocardial ischemia had higher BMI, METS-IR, and proportion of NAFLD. (II) The multivariate logistic regression analysis indicated that there was no significant correlation between MRTS-IR and myocardial ischemia. Furthermore, BMI and NAFLD were independent risk factors for myocardial ischemia. (III) METS-IR was independently positively correlated with NAFLD. As the METS-IR index increased, the risk of NAFLD significantly rose. (IV The results of the mediation analysis showed that NAFLD was a complete mediator variable between MERSIR and myocardial ischemia. This indicates that the effect of METS-IR on myocardial ischemia is completely mediated by NAFLD.

Globally, myocardial ischemia is the main cause of cardiovascular death, and the mortality rate of IHD is significantly higher than that of other types of cardiovascular diseases (2). Data indicate that patients with myocardial ischemia have a 13% probability of experiencing MACEs within 2 years (26). Therefore, it is important to identify the risk factors of myocardial ischemia and to pinpoint the precise targets for prevention and treatment.

In the future, along with the increase in obesity rates, the prevalence of obesity-related IR and CVD will also increase (9). Clinically, IR refers to a state where the sensitivity and responsiveness to insulin are reduced. It usually emerges several years before the onset of diabetes and has been proven to increase the risk of CVD (27). It has also been widely confirmed that insulin resistance has an adverse impact on the prognosis of CVDs (28, 29). METS-IR is a novel, easily accessible, and calculable non-insulin-based fasting IR index that has been used in research related to CVDs (30, 31). This research has confirmed that there is a significant correlation between METS-IR and myocardial ischemia. In line with these conclusions, there is increasing evidence that IR indices that are not based on insulin are linked to myocardial ischemia. Zhang et al. found the first evidence of a significant association between the TyG index and myocardial ischemia in individuals with ischemia and no obstructive CAD (14).

In our study, significant statistical differences were detected in EFV and FAV, and the incidence of abdominal obesity, DM, and NAFLD among the subgroups related to the tertiles of the METS-IR index. These results indicate that patients with abdominal obesity, DM, and NAFLD all have insulin resistance, which is consistent with previous studies (6, 32, 33). The results of this study indicate that METS-IR is independently positively correlated with NAFLD. As the METS-IR index increases, the risk of NAFLD significantly rises. This is similar to previous studies, as Zou et al. identified a positive association between the cardiometabolic index (CMI) and an elevated risk of NAFLD in the general population (34). The results also indicate that there is a correlation between METS-IR and both EFV and FAV. However, these relationships require further research to explore.

This study indicates that NAFLD serves as a bridge between METS-IR and myocardial ischemia. This indicates that insulin resistance, once it occurs after the development of NAFLD, significantly increases the risk of myocardial ischemia. NAFLD is the most common chronic liver disease globally (35) and approximately 38% of the population included in this study had NAFLD, which is consistent with previous reports. Previous studies have shown that NAFLD and myocardial ischemia are independently associated (36). NAFLD may influence myocardial ischemia directly or indirectly via mechanisms including insulin resistance, activation of the renin-angiotensin system and sympathetic nervous system, systemic inflammation, oxidative stress, and alterations in gut microbiota (37, 38). This study confirmed that IR may be the main pathway through which patients with NAFLD suffer from myocardial ischemia.

The association between METS-IR, a surrogate marker of IR, and myocardial ischemia is supported by pathophysiological mechanisms. In the presence of NAFLD, liver fat deposition triggers insulin resistance, leading to increased plasma free fatty acids. This exacerbates liver fat accumulation and induces myocardial insulin resistance, reducing glucose uptake by the heart and increasing fatty acid oxidation. Ultimately, this leads to inadequate energy supply to the myocardium, contributing to myocardial ischemia (39). Additionally, when the metabolism of insulin leads to the occurrence of NAFLD, fat accumulation in the liver leads to hepatocyte injury, activating inflammatory responses and releasing cytokines such as TNF-α and IL-6. These cytokines affect overall metabolism and vascular function. Inflammation also impairs vascular function and exacerbates myocardial ischemia (40). Therefore, NAFLD is a crucial factor in the development of myocardial ischemia caused by insulin resistance.

When the level of insulin resistance in the human body is relatively low, it is often difficult for clinicians to detect and pay sufficient attention to it clinically. This study confirms that NAFLD is an important bridge between IR and myocardial ischemia, providing a potential target and theoretical basis for the precise prevention and treatment of myocardial ischemia. Comprehensive management of patients with NAFLD, including lifestyle adjustments (such as diet control, weight management, and increased physical exercise) and pharmacological interventions (such as insulin sensitizers, antioxidants, and statins), can help reduce the risk of myocardial ischemia and MACEs (41). In summary, when IR exists, especially when it is accompanied by NAFLD, close clinical attention should be paid to these patients and early intervention measures should be taken to prevent the occurrence and development of myocardial ischemia.

### Limitations

This study possesses certain limitations that should be acknowledged. First, treatment variables that might influence METS-IR, such as the use of fibrates, statins, and specific oral antidiabetic drugs, which could impact insulin metabolic function, were not accounted for in the analysis. In the future, we will investigate the longitudinal effects of these drugs. Second, this study was a single-center investigation with a limited sample size, which may compromise the robustness and generalizability of the findings. In the future, we will expand the sample size to investigate differences in different subgroups. Finally, the scope of this research was confined to a correlation analysis without exploring patient outcomes or interventions targeting NAFLD, precluding an evaluation of any improvements in myocardial ischemia following treatment for fatty liver disease.

## Conclusion

This study provides evidence of the relationship between METS-IR and myocardial ischemia and highlights the important mediating role of NAFLD in this relationship. It provides new evidence and ideas for the precise prevention and treatment of myocardial ischemia.

## Data Availability

The raw data supporting the conclusions of this article will be made available by the authors, without undue reservation.
